# Impact of Prolonged Temporal Discrimination Threshold on Finger Movements of Parkinson’s Disease

**DOI:** 10.1371/journal.pone.0167034

**Published:** 2016-11-28

**Authors:** M. J. Lee, J. S. Son, J. H. Lee, S. J. Kim, C. H. Lyoo, M. S. Lee

**Affiliations:** 1 Department of Neurology, Pusan National University Hospital, Pusan National University School of Medicine and Biomedical Research Institute, Busan, Republic of Korea; 2 Department of Physical Medicine & Rehabilitation, Northwestern University, Chicago, Illinois, United States of America; 3 Department of Neurology, Pusan National University Yangsan Hospital, Pusan National University School of Medicine and Biomedical Research Institute, Yangsan, Republic of Korea; 4 Department of Neurology, Busan Baik Hospital, Inje University College of Medicine, Busan, Republic of Korea; 5 Department of Neurology, Gangnam Severance Hospital, Yonsei University College of Medicine, Seoul, Republic of Korea; University of Ottawa, CANADA

## Abstract

**Introduction:**

Sensory information is essential for the precise control of movement. Patients with Parkinson’s disease (PD) have higher-order sensory dysfunctions including prolonged temporal discrimination threshold (TDT). However, the impact of prolonged TDT on parkinsonian motor deficits is uncertain.

**Methods:**

This study includes 33 PD patients and 24 healthy controls. TDT values were measured in the index finger. Using coin rotation task (CRT), dexterous finger movement was assessed. Using an inertial sensor, the speed, amplitude, and frequency of finger tapping were measured. The impact of prolonged index finger TDT on two different finger movements was analyzed using the general estimating equation.

**Results:**

Compared to healthy controls, TDT was prolonged in the PD patients. There was no impact of TDT on mean values or decrement for amplitude and speed, as well as mean values, decrement and variability of tapping frequency. However, prolonged TDT had a significant impact on the variability in amplitude (B = 436.905 × 10^−4^, Wald *χ*^2^ = 9.140, *p* = 0.014) and speed (B = 425.655 × 10^−4^, Wald *χ*^2^ = 9.876, *p* = 0.014) of finger tapping. There was a marginal correlation between TDT and CRT. In addition, CRT correlated with variability in amplitude and speed of finger tapping.

**Conclusion:**

In PD, cutaneous temporal discriminative sensory dysfunction appears to be related to increased variabilities in the speed and amplitude of fast repetitive finger movements and disturbed finger dexterity.

## Introduction

Parkinson’s disease (PD) is primarily a motor disorder characterized by bradykinesia, rigidity, resting tremor and postural instability. However, approximately half of PD patients have various sensory symptoms [1]. Indeed, previous studies have reported higher-order sensory function abnormalities associated with PD, including static joint position sense, perception of movement, and temporal and spatial discrimination of somatosensory stimuli [2]. Such higher-order sensory dysfunctions can disturb motor program generation, motor learning, prediction of motor outcomes, and detection and correction of errors of ongoing movements [3].

The temporal discrimination threshold (TDT) is defined as the shortest time interval needed for two temporally-separated stimuli to be perceived as two events. Prior studies have reported that in PD patients, TDT is prolonged for visual, auditory and tactile stimuli [2,4–6].

Cutaneous sensory information, particularly for digits, is a critical component of proprioception [7] and is important for kinesthetic perception and stereognosis [8]. Therefore, prolonged TDT in PD patients may have an impact on parkinsonian motor deficits. Indeed, such prolongation in the index finger of PD patients has a significant impact on the dexterity of finger movements [5].

However, previous studies have reported inconsistent findings about the impact of prolonged TDT on parkinsonian motor deficits. Some studies have reported a correlation between TDT values and the King’s College Hospital PD rating scale scores, Unified Parkinson’s Disease Rating Scale (UPDRS) total motor scores, subscores for axial motor deficits, and gait freezing scores [4, 9, 10]. However, other studies found no correlations between index finger TDT values and UPDRS total motor scores and subscores for finger bradykinesia [2, 5, 6]. A possible explanation for such discrepancies may lie in the inherent limitations of clinical rating scales.

In the present study, we measured UPDRS scores for rapid repetitive finger tapping as well as opening and closing of the hand (UPDRS Items 23 and 24), and assessed rapid finger tapping using inertial sensors. We also evaluated impairment of finger dexterity using a coin rotation task (CRT). We then studied the impact of prolonged TDT on three different measurements for finger movement deficits in patients with PD.

## Methods

### 1. Subjects and clinical evaluation

We enrolled parkinsonian patients at the Movement Disorder Clinic of Pusan National University Hospital from January to October 2015. We included those patients who fulfilled the UK Brain Bank criteria for PD [11]. Subjects were excluded if they exhibited tremors, hand dystonia, or other physical problems that could interfere with motor tasks or TDT measurements. One of the authors (MJ Lee) conducted neurological examinations and excluded those subjects who showed abnormalities in primary sensory modalities such as sense of light touch, pain, temperature, position or vibration. We also excluded subjects who had abnormalities in nerve conduction velocity studies, or a cognitive function test (Mini-Mental State Exam score ≤ 26).

The completed study included 33 PD patients and 24 healthy controls, all of whom were right handed. Of the 33 PD patients, 24 had never received antiparkinson treatment, and the remainder had received treatment with levodopa and dopamine agonists. For the nine treated patients, we measured UPDRS score, CRT performance, kinematic parameters of finger tapping, and TDT during a practically-defined off period (following > 12 hours withdrawal of antiparkinsonian medications). In addition, we assessed finger movement of both hands using the summation of UPDRS scores for Item 23 (finger tapping) and 24 (opening and closing of the hand in rapid succession). The study protocol was approved by the Institutional Review Board of Pusan National University Hospital, and written informed consent was obtained from all subjects.

### 2. Measurement of TDT

TDT values were measured by stimulating the index finger with a bar containing two Ag-AgCl surface electrodes of 1 cm diameter, separated at 3 cm apart. After applying gel on the index finger, pairs of rectangular electrical pulses of 0.2-ms duration were delivered with a Keypoint electromyography unit (Keypoint G4 workstation, Tonsbakken, Denmark), through surface electrodes applied to the volar surface of the index finger of the left and right hands. Electrical stimuli were controlled by manipulating the magnitude of current (mA). Before measuring TDT values, we investigated the sensory threshold of each individual by delivering a series of stimuli at increasing intensity starting from 1 mA in 0.1 mA steps. The index finger was stimulated with three times the sensory threshold intensity.

The first inter-stimulus interval (ISI) between two pulses was 10 ms. ISI was increased by 10-ms increments until the subjects reported perception of the stimuli as two separate stimuli. Subsequently, three more pairs of stimuli were provided with 5-ms progressive increases in ISI. The TDT was considered to be valid when the subject consistently reported that two temporally separated stimuli were felt with an ISI longer than the threshold. The test was repeated until the subject reported two stimuli for the same ISI three times. The ISI was designated as the TDT [6].

### 3. Kinematic analysis of finger tapping

Kinematic analysis of finger tapping was conducted as reported previously [12]. Two inertial sensors (14.5 × 14.7 × 2.34 mm, 6.2 g, KinetiSense, Great Lakes Neurotechnologies, Inc., Cleveland, Ohio, USA) were attached to the distal phalanx of the index finger and thumb. To avoid bias arising from digit lengths, we incorporated signal data from gyroscopes. Subjects were instructed to tap their index finger on the pad of the thumb as quickly and widely as possible. Kinematic data were collected for 15 s, with three trials performed on each hand, separated by 60 s rest periods. An eye patch was applied for visual deprivation during the finger tapping trials.

We calculated the mean values for amplitude, speed and frequency over 15 s of finger tapping, and defined variability as the coefficient of variance (CoV). Decrements in amplitude, speed and frequency across finger tapping trials were obtained as the slope of the fitted linear regression line across the scatter plot of kinematic parameters against the tap cycle [12]. The mean values for amplitude, speed, frequency, variability, and decrement in kinematic parameters were taken as the average over three trials for each subject’s hand.

### 4. Coin rotation tasks

For the CRT, subjects were asked to rotate a nickel 360° using the thumb, forefinger, and middle finger of each hand as rapidly as possible for 10 s. If a coin was dropped, it was immediately returned to the subject while the clock continued to run. The coin rotation scores were calculated as reported previously: coin rotation score = half turns–[(coin drop × 0.1) × half turns] [5]. The CRT was performed three times for each hand with an inter-trial rest period of approximately 10 s. The median values for the CRT scores were calculated, and an eye patch was applied for visual deprivation.

### 5. Statistical analyses

Statistical analyses were performed using SPSS (Version 18.0; SPSS Inc., Chicago, IL, USA). Due to a non-Gaussian distribution (S1 File) and collinearity of the variables (S2 File), the results were analyzed by non-parametric tests. Differences in age (Mann-Whitney U test) and gender (*χ*^2^ test) were assessed between PD patients and controls. Differences in age and UPDRS motor scores between *de novo* and treated PD patients were compared using Mann-Whitney U test. We used the general estimating equation (GEE) modeling for comparison of kinematic parameters, TDT, and CR scores between PD and control groups. The impact of TDT prolongation on kinematic parameters was also investigated by GEE modeling. While performing GEE modeling, the structure of the correlation matrix was designated as independent, and the γ distribution with log link was selected as the response model for GEE (S3 File). To adjust for multiple testing, Benjamini-Hochberg corrected *p* values were used to correct significance tests. Statistical significance was defined as *p* < 0.05.

## Results

### 1. General characteristics and TDT values

The present study included 33 PD patients (mean age ± SD = 64.2 ± 9.3 years, range = 46 ~ 80; male:female = 22:11) and 24 healthy controls (mean age ± SD = 61.8 ± 4.6 years, range = 57 ~70; male:female = 12:12). There were no significant group differences in age or gender (age, Mann-Whitney U test, *p* = 0.175; gender, *χ*^2^ test, *p* = 0.205). The mean (± SD) disease duration was 25.4 (± 27.3) months (range = 3–138). In PD patients, the mean UPDRS total score was 21.3 (± 8.32) and the mean UPDRS score for finger movement was 2.85 (± 1.42).

*De novo* and treated PD patients showed no significant differences in mean (± SD) age (62.8 ± 8.7 *vs*. 67.9 ± 10.4; Mann-Whitney U test, *p* = 0.142), gender ratio (M:F; 17:7 *vs*. 5:4; Fisher’s exact test, *p* = 0.438), and UPDRS score (21.3 ± 8.3 *vs*. 22.2 ± 9.7; Mann-Whitney U test, *p* = 0.328). However, compared to *de novo* PD patients, treated PD patients had significantly longer disease duration (15.1 ± 10.8 vs.53.0 ± 38.4 months; Mann-Whitney U test, *p* = 0.001).

In comparisons of the PD patients and the controls, GEE analyses adjusted for age, gender and hand side revealed significantly prolonged TDT values in the PD patients than controls (PD 98.8 ± 37.6 msec; controls 53.8 ± 18.3 msec; B = -0.644, Wald *χ*^2^ = 89.582, corrected *p* < 0.001; Table 1). When the PD group was divided into *de novo* and treated groups, GEE modeling with the same covariate did not find a significant difference in TDT values between subgroups (*de novo* PD, 97.7 ± 38.7 msec; treated PD, 101.7 ± 35.2 msec, B = -0.27, Wald *χ*^2^ = 0.133, *p* = 0.716). Using disease duration, hand side, and subgroup (*de novo* and treated PD patients) as independent variables, GEE modeling showed that disease duration had no significant impact on TDT values (B = -0.002; Wald χ^2^ = 1.288, p = 0.256).

**Table 1:** Comparisons of kinematic parameters, coin rotation scores, and TDT values between PD and control groups.

	Left side		Right side		GEE		
	Control	PD	Control	PD	B (*Wald χ*^*2*^)	Mean difference (QICC)	*p*
**Mean values**^†^							
Amplitude (°)	86.70 ± 24.45	72.23 ± 21.15	83.24 ± 17.42	64.39 ± 22.48	0.052 (10.807)	0.224 (10.713)	0.002**
Speed (°/sec)	529.36 ± 164.73	393.98 ± 155.46	496.93 ± 104.46	347.47 ± 130.62	0.059 (18.817)	0.353 (10.459)	< 0.001**
Frequency (Hz)	3.05 ± 0.32	2.77 ± 0.62	3.04 ± 0.55	2.85 ± 0.73	0.104 (6.426)	0.109 (14.774)	0.014*
**Slope**^‡^							
Amplitude (°/cycle)	-0.08 ± 0.26	-0.38 ± 0.45	-0.14 ± 0.50	-0.31 ± 0.36	0.023 (8.614)	0.224 (10.195)	0.005**
Speed (°/sec/cycle)	-1.51 ± 1.67	-2.20 ± 2.13	-0.77 ± 2.10	-1.63 ± 2.01	0.102 (5.177)	0.863 (17.806)	0.025*
Frequency (Hz/cycle)	-0.01 ± 0.01	0.00 ± 0.01	0.00 ± 0.01	0.00 ± 0.02	0.102 (5.177)	-0.002 (10.000)	0.400
**Variability** (CoV, ×10^−2^)							
Amplitude	10.96 ± 4.94	18.71 ± 8.66	12.09 ± 6.02	17.10 ± 9.25	-0.432 (15.321)	-0.062 (34.400)	< 0.001**
Speed	11.44 ± 4.14	19.25 ± 8.44	11.14 ± 5.04	17.14 ± 9.17	-0.484 (24.538)	-0.071 (31.106)	< 0.001**
Frequency	7.46 ± 2.84	18.86 ± 19.57	7.35 ± 3.11	15.26 ± 15.22	-0.799 (24.959)	-0.091 (58.033)	< 0.001**
**CR score**	13.83 ± 2.68	9.89 ± 3.47	14.46 ± 3.49	10.76 ± 3.35	0.347 (36.429)	4.081 (18.132)	< 0.001**
**TDT** (msec)	53.75 ± 19.96	97.27 ± 38.16	53.75 ± 16.89	100.30 ± 37.50	-0.644 (89.582)	-48.391 (23.355)	< 0.001**

Mean ± SD; CR score = coin rotation score; TDT = temporal discrimination threshold; B = B-value in general estimating equation (GEE) model; Mean difference = difference of estimated marginal means, control—PD; QICC = goodness of model fit, corrected quasi-likelihood under independence model criterion; *p* = Benjamini-Hochberg corrected *p*-values for multiple testing;

^†^ = transformed by log function;

^‡^ = transformed by adding a constant;

* = *p* < 0.05;

** = *p* < 0.01.

### 2. Comparison of finger movement parameters between PD and control groups

The kinematic parameters of finger tapping and CRT performance for PD and control groups are summarized in Table 1 and S4 File. Compared to the controls, CRT performance by PD patients was significantly worse (PD *vs*. control; left side = 9.89 ± 3.47 *vs*.13.83 ± 2.68; right side = 10.76 ± 3.35 *vs*. 14.46 ± 3.49; *p* < 0.001). The PD group showed significantly reduced mean values for amplitude, speed and frequency. Additionally, PD patients showed greater decrement in amplitude and speed, and significantly higher variability in amplitude, speed, and frequency. The statistical significance for these remained after correction for multiple testing.

### 3. Impact of TDT prolongation on finger movement parameters

**Fig 1:**
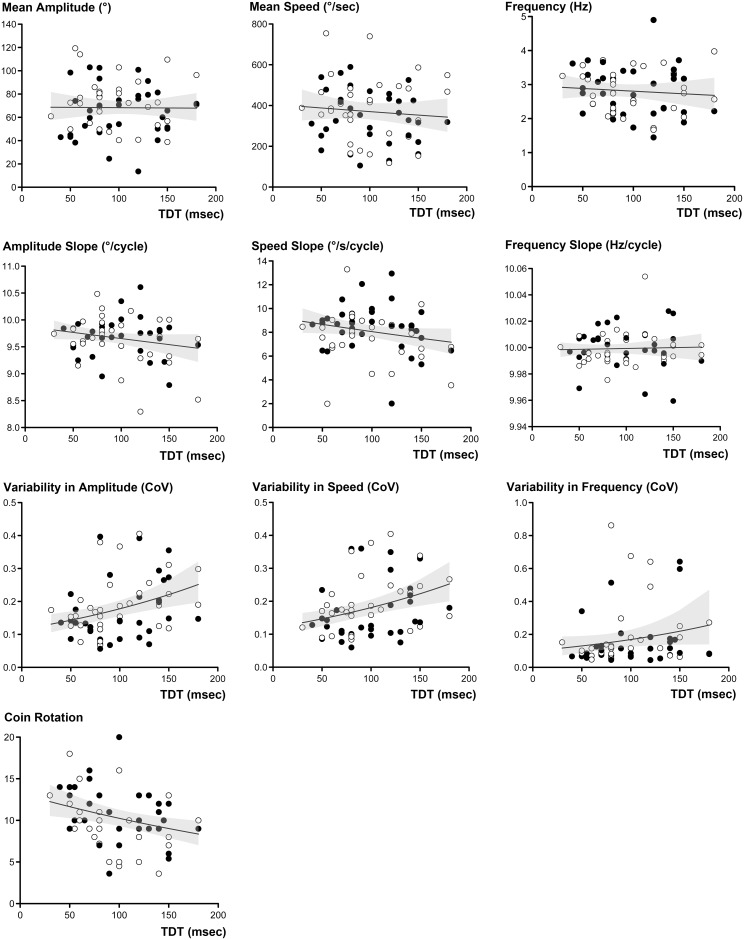
Scatter plots showing the relationship between TDT values and finger movement-related parameters in PD group. Slope-related parameters were transformed to positive values by adding a constant (+10). Open circles represent the left side hand and filled circles represent the right side hand. The fitted line represents the predicted mean response in the GEE model (predictor = TDT values, response = finger movement-related parameters), and the shaded area indicates 95% confidence interval. TDT = temporal discrimination threshold; CoV = coefficient of variance)

GEE analyses using age and hand side as covariates showed that TDT prolongation in PD patients had a marginally significant impact on UPDRS scores for finger movement (TDT, B = 36.039 × 10^−4^, Wald *χ*^2^ = 5.564, corrected *p* = 0.050). The TDT values for the PD patients had no significant influence on mean values or progressive decrement in amplitude, speed or frequency measured by the inertial sensors. However, TDT prolongation had a significant impact on variability in amplitude and speed (Amplitude CoV, B = 436.905 × 10^−4^, Wald *χ*^2^ = 9.140, corrected *p* = 0.014; speed CoV, B = 425.655 × 10^−4^, Wald *χ*^2^ = 9.876, corrected *p* = 0.014), but had no impact on frequency variability (Tables 2 and 3, Fig 1).

**Table 2:** Impact of TDT prolongation on kinematic parameters of finger tapping in PD patients.

	B (× 10^−4^)	SE (× 10^−4^)	*Wald χ*^*2*^	QICC	*p*
**Mean values**^†^					
Amplitude (°)	-1.636	2.463	0.441	8.556	0.985
Speed (°/sec)	-0.637	2.053	0.096	8.378	0.713
Frequency (Hz)	4.500	8.035	0.314	12.156	0.713
**Slope**^‡^					
Amplitude (°/cycle)	-2.286	1.274	3.217	8.113	0.134
Speed (°/s/cycle)	-15.597	7.430	4.406	13.559	0.079
Frequency (Hz/cycle)	-0.001	0.040	< 0.001	8.000	0.985
**Variability (CoV)**					
Amplitude	43.691	14.452	9.140	21.401	0.014*
Speed	42.566	13.545	9.876	20.564	0.014*
Frequency	54.509	33.733	2.611	46.633	0.167
**CR score**	-24.193	10.002	5.851	15.037	0.050
**UPDRS finger score**	36.039	15.279	5.564	21.082	0.050

TDT = temporal discrimination threshold; CoV = coefficient of variance; B = B value in general estimating equation (GEE) model; SE = standard error; QICC = goodness of model fit, corrected quasi likelihood under independence model criterion; *p* = Benjamini-Hochberg corrected *p*-values for multiple testing; CR = coin rotation; UPDRS = Unified Parkinson’s Disease Rating Scale;

^†^ = transformed by log function;

^‡^ = transformed by adding a constant;

* = *p* < 0.05

**Table 3:** Parameter estimates of GEE models in PD patients showing relationship between TDT values and variabilities in amplitude and speed.

	Response	Predictors	Parameter estimates				
			B (× 10^−4^)	SE (× 10^−4^)	95% Wald CI	Wald *χ*^*2*^	*p*
**GEE model 1**	Variability in amplitude (CoV)	Age	18.473	103.814	-0.018 ~ 0.022	0.032	0.859
		Hand side	1025.206	942.419	-0.082 ~ 0.287	1.183	0.277
		TDT	43.691	14.452	0.002 ~ 0.007	9.140	0.003*
**GEE model 2**	Variability in speed (CoV)	Age	5.876	92.587	-0.018 ~ 0.019	0.004	0.949
		Hand side	1318.765	944.472	-0.053 ~ 0.317	1.950	0.163
		TDT	42.565	13.545	0.002 ~ 0.007	9.876	0.002*

GEE = general estimating equation; TDT = temporal discrimination threshold; B = B values in GEE model; SE = standard error; CI = confidence interval; *p* = uncorrected *p*-values for multiple testing;

* = *p* < 0.05.

**Fig 2:**
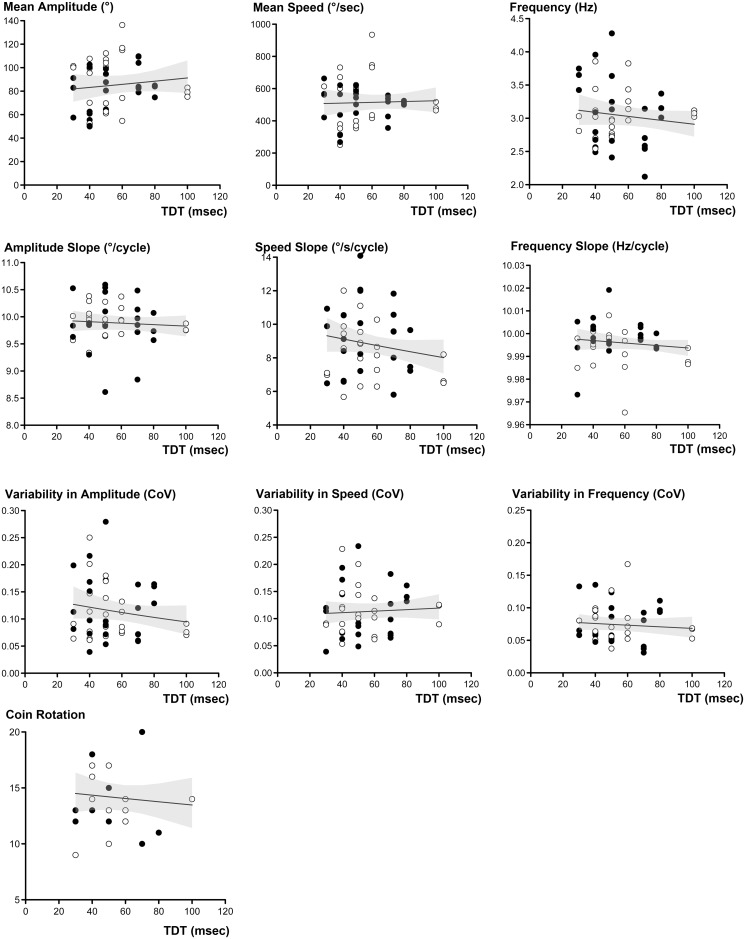
Scatter plots showing the relationship between TDT values and finger movement-related parameters in control group. Slope-related parameters were transformed to positive values by adding a constant (+10). Open circles represent the left side hand and filled circles represent the right side hand. The fitted line represents the predicted mean response in the GEE model (predictor = TDT values, response = finger movement-related parameters), and the shaded area indicates 95% confidence interval. TDT = temporal discrimination threshold; CoV = coefficient of variance)

In GEE analyses using age and hand side as covariates, decrements in amplitude had a significant impact on the variability in amplitude (B = -0.461, Wald *χ*^2^ = 15.881, *p* < 0.001). The impact of decrement on speed was also significant for the variability in speed (B = -0.058, Wald *χ*^2^ = 7.830, *p* = 0.005). We therefore performed additional GEE analyses including decrement in amplitude and speed as independent variables. GEE analysis using age, hand side, TDT values and decrement in amplitude as independent variables showed a significant impact of TDT values on the amplitude CoV (TDT, B = 0.004, Wald *χ*^2^ = 6.094, *p* = 0.014). After the same GEE analysis was conducted for speed, the impact of TDT on speed CoV remained significant (TDT, B = 0.004, Wald *χ*^2^ = 6.872, *p* = 0.009). For the control group, GEE analysis did not show a significant influence of TDT values on the kinematic parameters of finger tapping (Table 4 and Fig 2).

**Table 4:** Impact of TDT prolongation on kinematic parameters of finger tapping in the control group.

Kinematic parameters	B (× 10^−4^)	SE (× 10^−4^)	*Wald χ*^*2*^	*p*	QICC
**Mean values**^†^					
Amplitude	4.138	4.884	0.718	0.397	8.105
Speed	1.557	3.658	0.181	0.670	8.068
Frequency	-8.223	6.947	1.401	0.237	8.781
**Slope**^‡^					
Amplitude	-1.393	2.174	0.411	0.522	8.076
Speed	-21.389	13.479	2.518	0.113	10.020
Frequency	-0.050	0.052	0.943	0.332	8.000
**Variability**					
Amplitude	-32.752	24.296	1.817	0.178	14.795
Speed	21.991	20.601	1.140	0.286	14.074
Frequency	-18.015	18.750	0.923	0.337	14.656
**Coin rotation score**	-10.779	17.416	0.383	0.536	10.269

B = B value in general estimating equation (GEE) model; SE = standard error; *p* = uncorrected *p*-values for multiple testing; QICC = goodness of model fit, corrected quasi likelihood under independence model criterion;

^†^ = transformed by log function;

^‡^ = translated by adding a constant

GEE analysis covariated with age and hand side showed that TDT prolongation in the PD patients had a negative effect on CRT performance, however, the statistical significance was marginal (TDT, B = -24.193 × 10^−4^, Wald *χ*^2^ = 5.851, *p* = 0.050). In the control group, there was no significant correlation between TDT values and CRT performance (S4 File).

**Fig 3:**
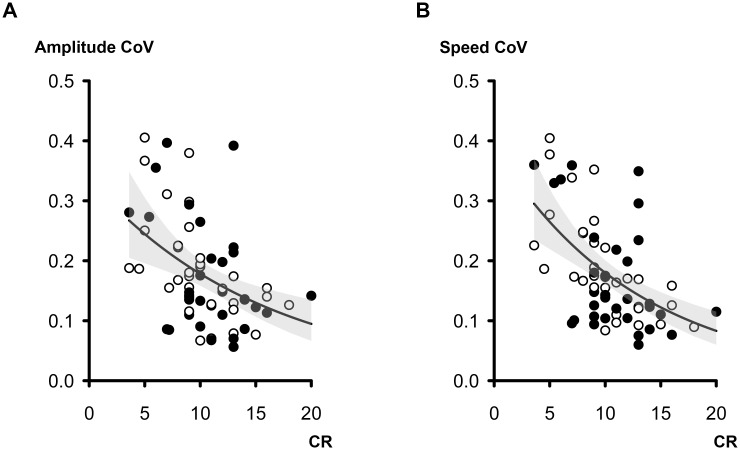
Scatter plots in the PD (A) and control (B) groups showing the relationship between CR scores and variabilities in amplitude and speed. Open circles represent the left side hand, and filled circles represent the right side hand. The fitted line represents the predicted mean response in the GEE model (predictor = TDT values, response = variabilities in amplitude and speed), and the shaded area indicates 95% confidence interval. CR = coin rotation; CoV = coefficient of variance.

Finally, we performed additional analyses investigating whether the increased variability in amplitude and speed might be associated with CRT performance. GEE analyses covariated with age and hand side revealed that variability in amplitude and speed had a significant negative impact on CRT performance (amplitude CoV, B = -1.780, Wald *χ*^2^ = 12.865, *p* < 0.001; speed CoV, B = -2.271, Wald *χ*^2^ = 20.355, *p* < 0.001; Fig 3).

## Discussion

In the present study, PD patients were found to have abnormally prolonged TDT. In comparison to the control group, gyroscopic analysis of finger tapping showed that these patients had significantly reduced mean values and higher variability in amplitude, speed and frequency. Decrements in amplitude and speed were more severe in the PD patients than the controls, but no significant difference was observed for decrement in frequency of finger tapping. In PD patients, prolonged TDT did not show significant correlation with the mean values or decrement in amplitude and speed, but did correlate with higher variability in amplitude and speed. In addition, TDT prolongation had a marginal negative correlation with CRT performance that correlated with variability in amplitude and speed of finger tapping.

### Pathogenesis of prolonged TDT in PD

In healthy subjects, administration of haloperidol causes overestimation of millisecond-level time intervals [13]. Levodopa treatment for PD patients is known to reduce prolonged TDT [2]. Dopamine transporter positron emission tomography (DAT PET) studies of PD patients have demonstrated a correlation between prolonged TDT and reduced striatal DAT binding [6]. Therefore, it is likely that the processing of brief durations in the range of milliseconds appears to be modulated by dopaminergic activity in the basal ganglia [13].

The present study showed that prolongation of TDT occurs from the early stage of PD when pathological lesions are more or less confined to the nigral dopaminergic neurons [14]. Therefore, in PD, it could be possible that striatal dopamine loss might affect temporal processing of external sensory inputs and cause TDT prolongation [9].

A large proportion of caudate neurons receives proprioceptive [15] and discriminative cutaneous sensory inputs [16, 17]. In monkeys, a microelectrode recording study reported that caudate neuronal response to sensory stimuli becomes less active after 1-methyl-4-phenyl-1,2,3,6-tetrahydropyridine (MPTP)-treatment [18]. It has been proposed that basal ganglia circuits are interconnected in an open-loop manner [19]. Thus, sensory information processed in the associative basal ganglia circuit, connecting the prefrontal cortex and caudate nucleus, may participate in the monitoring and modulation of movement occurring in the motor and motor-related cortical areas [20].

In PD patients, increased variability in amplitude and speed of finger tapping correlates with reduced DAT binding of the caudate nucleus, but not of the putamen [12]. We suspect that impairment of higher-order sensory processing in the dopamine deprived striatum, particularly in the caudate, increases variability of amplitude and speed of finger tapping.

### Prolonged TDT and increased variability in amplitude and speed of finger tapping

Precise sensory input is essential for predicting motor outcome and monitoring of ongoing movement. Tactile sensation in the digit is a major component of proprioception and kinesthesia [7, 8]. Thus TDT prolongation of the digit in PD might reflect impaired proprioceptional sensation [5]. Finger tapping is a very fast movement using on-line feedback, and is mainly controlled by a predictive feed-forward mechanism [3, 21]. However, the motor program for upcoming tapping is recalibrated by the kinematic parameters of ongoing tapping [3, 21, 22].

If sensory feedback becomes inaccurate, so too does the predictive feed-forward mechanism [23, 24]. Interestingly, kinematic analyses of keyboard typing in healthy subjects showed that blockade of tactile sensation in the fingertips reduces accuracy and increases performance variability during rapid fine movement [25, 26]. Accordingly, our observations suggest that defective processing of somatosensory inputs is associated with failure to maintain amplitude and speed of fast finger tapping.

### TDT prolongation and mean amplitude, speed and frequency-related parameters of finger tapping

It has been suggested that defective sensory processing is a factor influencing the severity of bradykinesia in PD patients [2]. In a study of PD, prolonged TDT correlated with reaction time and movement time of a single ballistic wrist movement [4].

Motor basal ganglia circuit, connecting the motor cortex, premotor cortex, SMA and posterior putamen, is the primary determinants of amplitude and speed of movement [27]. In a DAT PET study, the mean values for amplitude and speed of finger tapping correlated most closely with DAT binding in the putamen [12].

In the present study, TDT prolongation in PD patients did not have a significant correlation with progressive decrement in amplitude and speed (the so called ‘sequence effect’), or frequency-related parameters. Although the precise mechanism of the sequence effect is not clear yet, it seems to be related to dysfunction of the non-dopaminergic pathway outside the basal ganglia [12, 28].

In a previous DAT PET study, striatal dopamine loss did not correlate with the mean values, decrement or variability of frequency of finger tapping [12]. The present study showed no significant difference in the maximal frequency of finger tapping between the PD patients and healthy controls. Patients with early PD have been shown to increase gait cadence to compensate for reduced stride length [29]. Taken together, these findings suggest that the pulse generator for the maximal frequency of repetitive rhythmic movement is not affected by striatal dopamine loss and is probably located outside of the basal ganglia (e.g. cerebellum) [12, 30].

### Coin rotation and UPDRS finger bradykinesia scores

Discriminative cutaneous sensory information arising from the glabrous skin is important for the control of dexterous finger movement [5,31]. In the present study, TDT prolongation showed a marginally significant correlation with poor CRT performance, and variability in amplitude and speed was negatively correlated with CRT performance. These suggest that the defective sensory processing might serve as a common pathophysiologic background for prolonged TDT, poor CRT performance, and increased variability in amplitude and speed of finger tapping.

In contrast to a previous report, the present study showed a marginally significant correlation between prolonged TDT and UPDRS scores for finger movement [5]. The discrepancy may be due to limitations of the clinical rating scale (UPDRS) or the differences in disease severity of PD patients included in the studies (UPDRS total score of the present study = 21.30 ± 8.32 and the previous study = 31.4 ± 11.5). Compared to the previous study, PD patients in the present study had a narrower range of UPDRS scores for finger movement (present study 2.85 ± 1.42 *vs*. previous study 3.3 ± 1.6). In addition, the previous study evaluated the TDT and UPDRS finger score only of the dominant hand.

### Limitations

We acknowledge several limitations in the present study. First, we excluded tremor-dominant PD patients for accurate TDT measurement and kinematic analysis of finger tapping. Therefore, it is unclear whether TDT prolongation also has a similar impact on kinematic parameters of finger movement in tremor-dominant PD patients. Second, we did not perform neuropsychological tests to assess the direct impact of executive or attentional dysfunction on TDT prolongation, increased variability of the kinematic parameters of finger tapping or dexterous finger movement. Third, we increased ISI linearly rather than in a randomized fashion, and this might cause an overestimation of TDT values [4]. Fourth, we did not employ systematic approach to assess primary sensory function.

## Conclusions

In the present study, prolonged TDT in PD patients showed a significant correlation with the increased variabilities in amplitude and speed of finger tapping. Therefore, in PD, the disturbance in the processing of temporal elements of cutaneous tactile sensation of the digits is likely to be one of possible factors contributing to the inconsistency of fast repetitive finger movement. Such higher order sensory dysfunction may also contribute to impairment of finger movement dexterity that are important for activities of daily living.

## Supporting Information

S1 File Results of normality tests. Histograms of the variables and results of Kolmogorov-Smirnova and Shapiro-Wilk tests in PD and control groups.
(DOCX)

S2 File Pairwise scatter plots between the variables. (DOCX)

S3 File Gamma quantile-quantile plots of the variables in PD and control groups. (DOCX)

S4 File Descriptive statistics of PD and control groups. (DOCX)

S5 File The dataset of the present study. (XLSX)
