# Supplementary material for: Impact of Prolonged Temporal Discrimination Threshold on Finger Movements of Parkinson’s Disease
Source: PLoS One. 2016 Nov 28;11(11):e0167034. doi: 10.1371/journal.pone.0167034 (PMC5125668; doi:10.1371/journal.pone.0167034)
Supplement: S1 File — Histograms of the variables and results of Kolmogorov-Smirnova and Shapiro-Wilk tests in PD and control groups. (DOCX) [file pone.0167034.s001.docx]

**Supplementary Material 1**

**1.1 Histograms of the variable in PD group**





**Abbreviations**: CoV = coefficient of variance; TDT = temporal discrimination threshold

**Units of the measurement**: Amplitude (°), Speed (°/sec), Frequency (Hz), Slope: amplitude (°/cycle), Slope: speed (°/sec/cycle), Slope: frequency (Hz/cycle), CoV-related values (CoV), TDT (msec)

**1.2 Histograms of the variable in control group**

**

**

**Abbreviations**: CoV = coefficient of variance; TDT = temporal discrimination threshold

**Units of the measurement**: Amplitude (°), Speed (°/sec), Frequency (Hz), Slope: amplitude (°/cycle), Slope: speed (°/sec/cycle), Slope: frequency (Hz/cycle), CoV-related values (CoV), TDT (msec)

**1.3. Testing normality of kinematic parameters, TDT, and CR score in PD and control groups**

|  | **PD** | | | | |  | **Control** | | | | |
| --- | --- | --- | --- | --- | --- | --- | --- | --- | --- | --- | --- |
|  | **Kolmogorov-Smirnova** | |  | **Shapiro-Wilk** | |  | **Kolmogorov-Smirnova** | |  | **Shapiro-Wilk** | |
|  | **Statistics** | ***p*** |  | **Statistics** | ***p*** |  | **Statistics** | ***p*** |  | **Statistics** | ***p*** |
| **Mean amplitude** | 0.074 | 0.200 |  | 0.986 | 0.646 |  | 0.101 | 0.200 |  | 0.966 | 0.173 |
| **Mean speed** | 0.058 | 0.200 |  | 0.977 | 0.272 |  | 0.086 | 0.200 |  | 0.975 | 0.377 |
| **Mean frequency** | 0.087 | 0.200 |  | 0.974 | 0.172 |  | 0.135 | 0.028**^*^** |  | 0.965 | 0.168 |
| **Amplitude slope** | 0.129 | 0.008**^**^** |  | 0.945 | 0.005**^**^** |  | 0.125 | 0.056 |  | 0.934 | 0.010**^*^** |
| **Speed slope** | 0.108 | 0.052 |  | 0.946 | 0.006**^**^** |  | 0.087 | 0.200 |  | 0.970 | 0.256 |
| **Frequency slope** | 0.111 | 0.041**^*^** |  | 0.933 | 0.002**^**^** |  | 0.163 | 0.003**^**^** |  | 0.895 | < 0.001**^**^** |
| **Amplitude CoV** | 0.148 | 0.001**^**^** |  | 0.904 | < 0.001**^**^** |  | 0.169 | 0.002**^**^** |  | 0.898 | 0.001**^**^** |
| **Speed CoV** | 0.150 | 0.001**^**^** |  | 0.902 | < 0.001**^**^** |  | 0.097 | 0.200 |  | 0.946 | 0.027 |
| **Frequency CoV** | 0.285 | < 0.001**^**^** |  | 0.648 | < 0.001**^**^** |  | 0.144 | 0.014**^*^** |  | 0.918 | 0.002**^**^** |
| **Coin rotation score** | 0.114 | 0.034**^*^** |  | 0.977 | 0.253 |  | 0.114 | 0.158 |  | 0.955 | 0.064 |
| **TDT** | 0.161 | < 0.001**^*^** |  | 0.953 | 0.013**^*^** |  | 0.227 | < 0.001**^**^** |  | 0.886 | < 0.001**^**^** |

* = *p* < 0.05; ** = *p* < 0.01
