# Supplementary material for: Impact of Prolonged Temporal Discrimination Threshold on Finger Movements of Parkinson’s Disease
Source: PLoS One. 2016 Nov 28;11(11):e0167034. doi: 10.1371/journal.pone.0167034 (PMC5125668; doi:10.1371/journal.pone.0167034)
Supplement: S2 File — (DOCX) [file pone.0167034.s002.docx]

**Supplementary Material 2**

**2.1 Pairwise scatter plots between the variables in PD group**


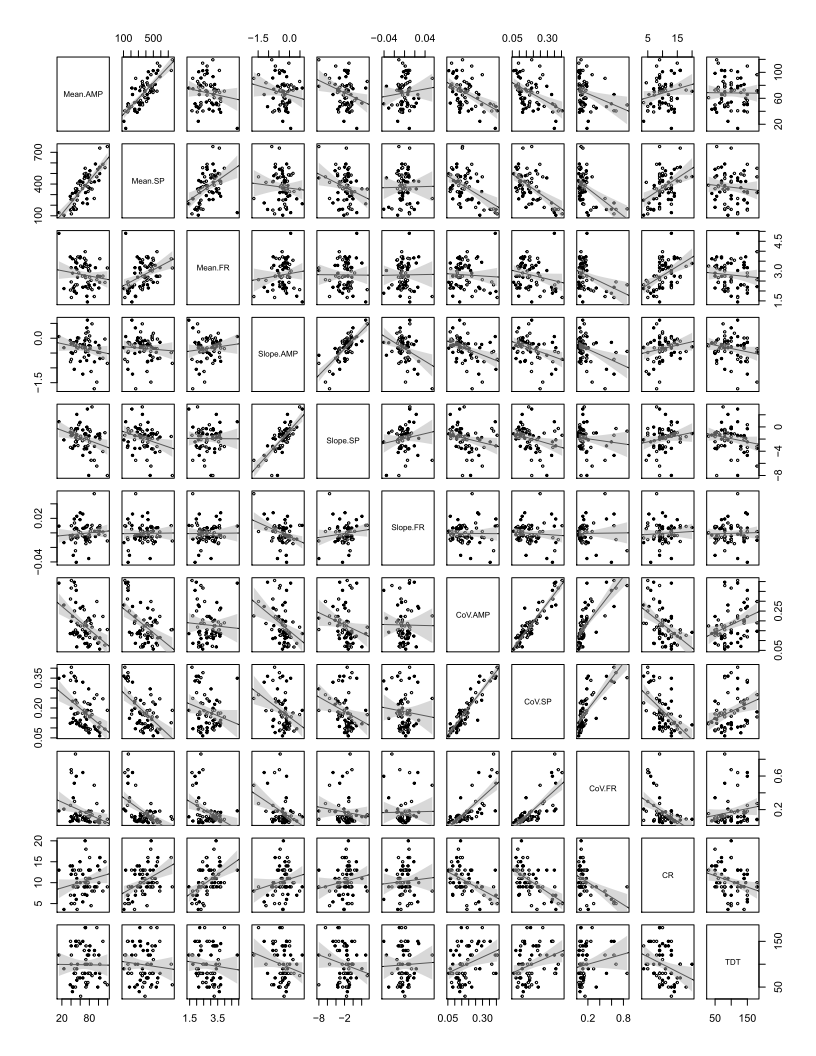


Open circles represent the left side hand, and filled circles represent the right side hand. The fitted line represents linear regression model fit, and the shaded area represents 95% confidence interval. *Abbreviations*: AMP = amplitude; SP = speed; FR = frequency; CoV = coefficient of variance; CR = coin rotation; TDT = temporal discrimination threshold

**2.2 Pairwise scatter plots between the variables in control group**


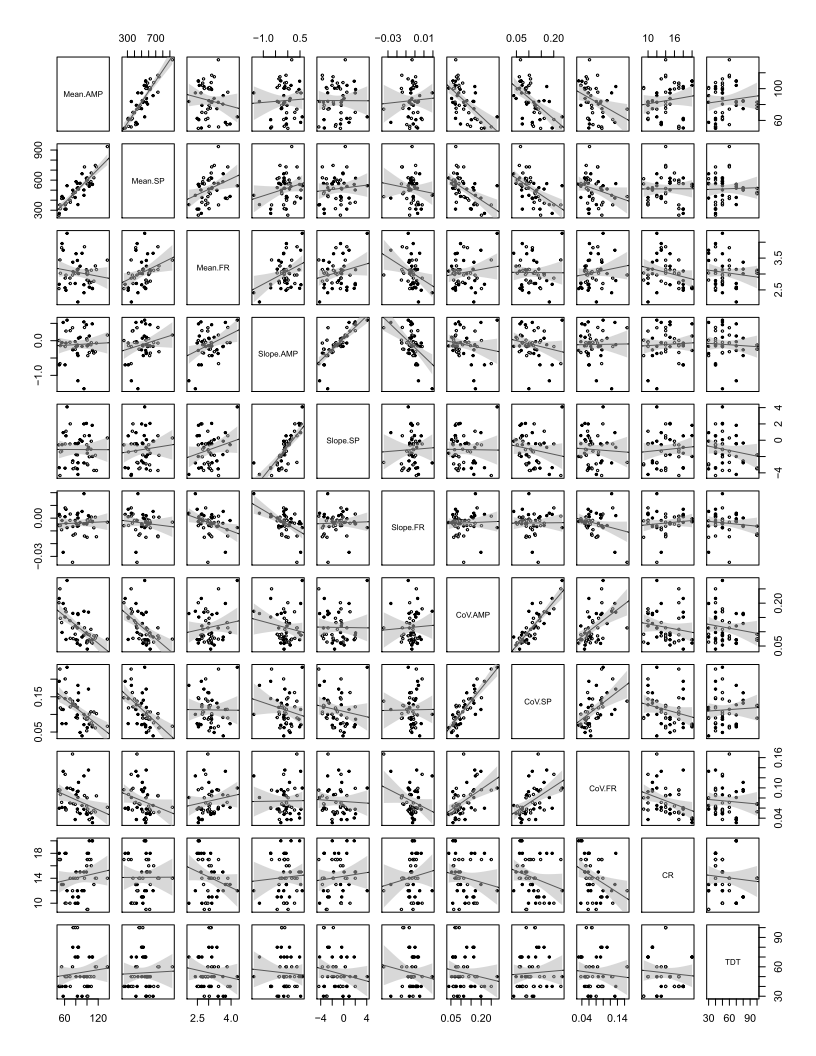


Open circles represent the left side hand, and filled circles represent the right side hand. The fitted line represents linear regression model fit, and the shaded area represents 95% confidence interval. *Abbreviations*: AMP = amplitude; SP = speed; FR = frequency; CoV = coefficient of variance; CR = coin rotation; TDT = temporal discrimination threshold

**Units of the measurement**: Amplitude (°), Speed (°/sec), Frequency (Hz), Slope: amplitude (°/cycle), Slope: speed (°/sec/cycle), Slope: frequency (Hz/cycle), CoV-related values (CoV), TDT (msec)

**2.3 Tests on multi-collinearity in kinematic parameters of PD patients**

**2.3.1 Collinearity statistics**

|  | **Tolerance** | **VIF** |
| --- | --- | --- |
| (constant) |  |  |
| Mean amplitude | 0.063 | 15.810 |
| Mean speed | 0.051 | 19.494 |
| Mean frequency | 0.134 | 7.472 |
| Amplitude slope | 0.098 | 10.173 |
| Speed slope | 0.110 | 9.111 |
| Frequency slope | 0.293 | 3.410 |
| Amplitude CoV | 0.084 | 11.941 |
| Speeed CoV | 0.076 | 13.135 |
| Frequency CoV | 0.232 | 4.313 |

Result of multicollinearity test from linear regression; CoV = coefficient of variance; VIF = variance inflation factor

**2.3.2 Multi-collinearity diagnostics for kinematic parameters of PD patients**

| Dimension | Eigen  value | Condition  index | Variance proportions | | | | | | | | | |
| --- | --- | --- | --- | --- | --- | --- | --- | --- | --- | --- | --- | --- |
|  |  |  | (constant) | Mean  amplitude | Mean  speed | Mean  frequency | Amplitude  slope | Speed  slope | Frequency  slope | Amplitude  CoV | Speed  CoV | Frequency  CoV |
| 1 | 7.184 | 1.000 | 0.09 | 0.1 | 0.11 | 0.12 | 0.7 | 0.75 | 0.01 | 0.28 | 0.25 | 1.43 |
| 2 | 1.120 | 2.532 | 0.03 | 0.01 | 0.04 | 0.05 | 7.42 | 0 | 216.3 | 0 | 0.02 | 0.34 |
| 3 | 0.792 | 3.011 | 0.26 | 0.91 | 1.66 | 0.76 | 4.02 | 1 | 17.56 | 1.16 | 1.09 | 47.84 |
| 4 | 0.657 | 3.306 | 0.25 | 0 | 0.03 | 0.27 | 18.61 | 42.78 | 20.17 | 1.35 | 0.96 | 18.82 |
| 5 | 0.133 | 7.346 | 0.03 | 9.74 | 2.5 | 5.93 | 3.47 | 11.8 | 16.6 | 17.94 | 12.71 | 276.07 |
| 6 | 0.051 | 11.896 | 0.13 | 19.85 | 2.22 | 45.23 | 184.98 | 90.08 | 143.82 | 0.64 | 54.47 | 59.63 |
| 7 | 0.031 | 15.127 | 18.58 | 9.25 | 14.85 | 13.18 | 532.4 | 436.18 | 387.03 | 2.15 | 6.07 | 471.61 |
| 8 | 0.023 | 17.859 | 78.52 | 1.23 | 66.47 | 16.14 | 26.15 | 122.28 | 36.74 | 216.23 | 0.43 | 106.98 |
| 9 | 0.007 | 31.332 | 34.24 | 31.35 | 101.18 | 1.24 | 168.8 | 243.76 | 45.97 | 704.67 | 846.01 | 2.22 |
| 10 | 0.002 | 64.975 | 867.86 | 927.55 | 810.93 | 917.08 | 53.45 | 51.36 | 115.8 | 55.56 | 77.99 | 15.07 |

Result of multi-collinearity test from linear regression; CoV = coefficient of variance
