# Supplementary material for: Impact of Prolonged Temporal Discrimination Threshold on Finger Movements of Parkinson’s Disease
Source: PLoS One. 2016 Nov 28;11(11):e0167034. doi: 10.1371/journal.pone.0167034 (PMC5125668; doi:10.1371/journal.pone.0167034)
Supplement: S3 File — (DOCX) [file pone.0167034.s003.docx]

**Supplementary material 3**

**3.1 Gamma Q-Q plots in PD group**





Before assessing gamma quantiles, slope-related parameters were transformed to positive values by adding a constant (+10). **Abbreviations**: CoV = coefficient of variance; TDT = temporal discrimination threshold **Units of the measurement**: Amplitude (°), Speed (°/sec), Frequency (Hz), Slope: amplitude (°/cycle), Slope: speed (°/sec/cycle), Slope: frequency (Hz/cycle), CoV-related values (CoV), TDT (msec)

**3.1 Gamma Q-Q plots in control group**





Before assessing gamma quantiles, slope-related parameters were transformed to positive values by adding a constant (+10). **Abbreviations**: CoV = coefficient of variance; TDT = temporal discrimination threshold **Units of the measurement**: Amplitude (°), Speed (°/sec), Frequency (Hz), Slope: amplitude (°/cycle), Slope: speed (°/sec/cycle), Slope: frequency (Hz/cycle), CoV-related values (CoV), TDT (msec)
