# Supplementary material for: Impact of Prolonged Temporal Discrimination Threshold on Finger Movements of Parkinson’s Disease
Source: PLoS One. 2016 Nov 28;11(11):e0167034. doi: 10.1371/journal.pone.0167034 (PMC5125668; doi:10.1371/journal.pone.0167034)
Supplement: S4 File — (DOCX) [file pone.0167034.s004.docx]

**Supplementary Material 4. Descriptive statistics for the control and PD groups**

**Control group, the left side**

|  | Means | SD | Median | 25% quantile | 75% quantile | Min | Max |
| --- | --- | --- | --- | --- | --- | --- | --- |
| **Mean amplitude** | 86.70 | 24.45 | 89.28 | 66.13 | 105.60 | 49.95 | 136.39 |
| **Mean speed** | 529.36 | 164.73 | 540.10 | 389.30 | 618.81 | 250.59 | 934.83 |
| **Mean frequency** | 3.05 | 0.32 | 3.02 | 2.84 | 3.13 | 2.54 | 3.86 |
| **Amplitude slope** | -0.08 | 0.26 | -0.09 | -0.25 | 0.04 | -0.66 | 0.38 |
| **Speed slope** | -1.51 | 1.67 | -1.39 | -2.96 | -0.46 | -4.33 | 2.01 |
| **Frequency slope** | -0.01 | 0.01 | 0.00 | -0.01 | 0.00 | -0.03 | 0.01 |
| **Amplitude CoV** | 0.11 | 0.05 | 0.09 | 0.07 | 0.14 | 0.06 | 0.25 |
| **Speed CoV** | 0.11 | 0.04 | 0.11 | 0.09 | 0.13 | 0.05 | 0.23 |
| **Frequency CoV** | 0.07 | 0.03 | 0.07 | 0.05 | 0.09 | 0.04 | 0.17 |
| **Coin rotation score** | 13.83 | 2.68 | 14.00 | 12.50 | 16.50 | 9.00 | 17.00 |
| **TDT** | 53.75 | 19.96 | 50.00 | 40.00 | 60.00 | 30.00 | 100.00 |

SD: standard deviation

**Control group, the right side**

|  | Means | SD | Median | 25% quantile | 75% quantile | Min | Max |
| --- | --- | --- | --- | --- | --- | --- | --- |
| **Mean amplitude** | 83.24 | 17.42 | 83.92 | 69.62 | 96.82 | 50.46 | 109.78 |
| **Mean speed** | 496.93 | 104.46 | 522.17 | 432.34 | 566.38 | 268.39 | 663.27 |
| **Mean frequency** | 3.04 | 0.55 | 3.05 | 2.58 | 3.40 | 2.12 | 4.28 |
| **Amplitude slope** | -0.14 | 0.50 | -0.13 | -0.33 | 0.12 | -1.39 | 0.59 |
| **Speed slope** | -0.77 | 2.10 | -0.49 | -2.65 | 0.55 | -4.19 | 4.09 |
| **Frequency slope** | 0.00 | 0.01 | 0.00 | 0.00 | 0.00 | -0.03 | 0.02 |
| **Amplitude CoV** | 0.12 | 0.06 | 0.11 | 0.07 | 0.16 | 0.04 | 0.28 |
| **Speed CoV** | 0.11 | 0.05 | 0.10 | 0.07 | 0.14 | 0.04 | 0.23 |
| **Frequency CoV** | 0.07 | 0.03 | 0.06 | 0.05 | 0.10 | 0.03 | 0.14 |
| **Coin rotation score** | 14.46 | 3.49 | 14.00 | 11.50 | 18.00 | 10.00 | 20.00 |
| **TDT** | 53.75 | 16.89 | 50.00 | 40.00 | 70.00 | 30.00 | 80.00 |

SD: standard deviation

**PD group, left side**

|  | Means | SD | Median | 25% quantile | 75% quantile | Min | Max |
| --- | --- | --- | --- | --- | --- | --- | --- |
| **Mean amplitude** | 72.23 | 21.15 | 72.46 | 55.11 | 82.11 | 38.90 | 119.36 |
| **Mean speed** | 393.98 | 155.46 | 389.09 | 327.84 | 484.55 | 118.28 | 754.48 |
| **Mean frequency** | 2.77 | 0.62 | 2.69 | 2.31 | 3.25 | 1.67 | 3.98 |
| **Amplitude slope** | -0.38 | 0.45 | -0.33 | -0.48 | -0.10 | -1.70 | 0.49 |
| **Speed slope** | -2.20 | 2.13 | -1.91 | -3.23 | -0.96 | -8.01 | 3.30 |
| **Frequency slope** | 0.00 | 0.01 | 0.00 | -0.01 | 0.01 | -0.02 | 0.05 |
| **Amplitude CoV** | 0.19 | 0.09 | 0.17 | 0.13 | 0.22 | 0.07 | 0.41 |
| **Speed CoV** | 0.19 | 0.08 | 0.17 | 0.13 | 0.23 | 0.08 | 0.40 |
| **Frequency CoV** | 0.19 | 0.20 | 0.12 | 0.08 | 0.18 | 0.05 | 0.86 |
| **Coin rotation score** | 9.89 | 3.47 | 9.00 | 8.00 | 12.00 | 3.60 | 18.00 |
| **TDT** | 97.27 | 38.16 | 80.00 | 75.00 | 120.00 | 30.00 | 180.00 |

SD: standard deviation

**PD group, right side**

|  | Means | SD | Median | 25% quantile | 75% quantile | Min | Max |
| --- | --- | --- | --- | --- | --- | --- | --- |
| **Mean amplitude** | 64.39 | 22.48 | 65.82 | 50.22 | 78.58 | 13.63 | 103.03 |
| **Mean speed** | 347.47 | 130.62 | 328.38 | 254.19 | 433.47 | 105.75 | 589.57 |
| **Mean frequency** | 2.85 | 0.73 | 2.90 | 2.22 | 3.29 | 1.45 | 4.90 |
| **Amplitude slope** | -0.31 | 0.36 | -0.24 | -0.48 | -0.14 | -1.21 | 0.61 |
| **Speed slope** | -1.63 | 2.01 | -1.46 | -2.47 | -0.84 | -7.99 | 2.95 |
| **Frequency slope** | 0.00 | 0.02 | 0.00 | -0.01 | 0.01 | -0.04 | 0.03 |
| **Amplitude CoV** | 0.17 | 0.09 | 0.14 | 0.11 | 0.21 | 0.06 | 0.40 |
| **Speed CoV** | 0.17 | 0.09 | 0.14 | 0.10 | 0.22 | 0.06 | 0.36 |
| **Frequency CoV** | 0.15 | 0.15 | 0.09 | 0.07 | 0.17 | 0.04 | 0.64 |
| **Coin rotation score** | 10.76 | 3.35 | 10.00 | 9.00 | 13.00 | 3.60 | 20.00 |
| **TDT** | 100.30 | 37.50 | 100.00 | 70.00 | 130.00 | 40.00 | 180.00 |

SD: standard deviation
